# Response to a Professor, and a Speculation upon the Sensorium

**Published:** 1850-07

**Authors:** 


### ARTICLE IV.

*A Response to a Professor, and a speculation upon the sensorum*: By BENNETT DOWLER, M. D. (From the author.)

The title of the pamphlet of sixteen pages, given above, might lead some to expect a severe repartee to the complaint of Prof. Le Conte, that Dr. Dowler, in his "Contributions to Physiology," neglected to give due credit to his published experiments upon the alligator. But a perusal of it discovers a plausible reason for not mentioning Dr. Le Conte's experiments in said paper, though he regrets now he had not strengthened his conclusions, by quoting them again. The explanation is simply this: Dr. Dowler had but a short time previously given a synopsis of Prof. Le Conte's experiments in the same periodical, the N. O. Med. Journal, which were in many respects similar to his own, and a want of room prevented their being re-produced.

A perusal of the pamphlet shows entire good feeling and a stronger preference for the development of truth and the establishment of the author's views of the physiology of the nerves and brain—of the seat of the sensorium, than a disposition to contend for priority; observing, "it is verity rather than priority, which is most important." The arguments in favor of the existence of mental power in other parts of the nervous system than the brain are plausible, at least. E.

